# Sociopolitical development of the nursing profession in Iran: a historical review

**Published:** 2016-11-05

**Authors:** Afsaneh Raiesifar, Mohammadreza Firouzkouhi, Marjaneh Fooladi, Soroor Parvizy

**Affiliations:** 1 PhD Candidate in Nursing, School of Nursing and Midwifery, Tehran University of Medical Sciences, Tehran, Iran;; 2 Assistant Professor, Department of Medical Surgical Nursing, Faculty of Nursing and Midwifery, Zabol University of Medical Sciences, Zabol, Iran;; 3 Professor, Faculty of Nursing, University of Jordan, Amman, Jordan;; 4 Professor, Department of Paediatric Nursing, Faculty of Nursing and Midwifery, Tehran University of Medical Sciences, Tehran, Iran; Center for Educational Research in Medical Sciences (CERMS), Iran University of Medical Sciences, Tehran, Iran.

**Keywords:** *Historical research*, *Iran-Iraq war*, *Islamic Revolution*, *Nursing history*, *White Revolution*

## Abstract

Significant sociopolitical changes in recent decades have not only influenced the nursing profession, but also the entire Iranian healthcare system. This study describes the historical evolution of the nursing profession within a sociopolitical context.

This historical review of unpublished and published literature endorsed personal accounts of historic events by 14 of the oldest nurses in Iran chosen through purposive sampling method, as they shared their nursing experiences. Individual recollections were collected through in-depth and semi-structured interviews and later analyzed through oral history analysis method.

From the results, the 3 categories of the White Revolution, the Islamic Revolution, and Iran-Iraq war and 8 subcategories emerged, where participants identified factors that fundamentally changed the Iranian nursing profession.

The nursing profession continues to develop and help revise policies to improve the healthcare system and quality of care. The findings of this study facilitate the better understanding of the influence of sociopolitical events on the nursing profession and guide the revision or development of new healthcare policies.

## Introduction

Historically, the nursing profession has been formed with complex ethical and social issues. Nursing discipline established upon its’ known historic patterns, and developed based on new sciences, medical innovations, and social movements (1). Over time, nursing has undergone considerable changes affecting the healthcare services and treatment modalities (2), and thus far, it has always been responsive to the ever-changing needs of patients and healthcare systems (3). In fact, nursing has gone through a unique historical process before reaching its current state as a trustworthy profession. 

New trends in the nursing profession have stemmed from numerous factors, such as wars, economic downturns, scientific progress, and nursing shortage. Nevertheless, nurses have continually played an important role in shaping the healthcare system (2). Since its inception, the nursing profession has faced various upheavals due to historical, religious, cultural, and economical factors (4). 

The history of nursing in Iran shows utilization of housewives, untrained hospital and clinic personnel, along with few trained nurses providing patient care before the establishment of academic nursing in 1915 (5). Similar to China, Taiwan, and Lebanon, the modern nursing program in Iran was modeled after the Christian missionaries who promoted religion while providing humanitarian nursing services (5). Missionaries provided healthcare services to local residents, and later, trained Iranian women as hospital nurses to fulfil their religious responsibilities. Nurses who graduated from the missionary nursing schools had to care for a large number of patients in the hospitals, and due to cultural barriers, poor social image, and demanding hours, few Iranian families allowed their daughters to enroll in the nursing programs (6). 

In 1935, and during the social movement of the Pahlavi dynasty, American nurse educators were recruited to establish the Iranian nursing programs in Tehran, Tabriz, and Mashhad (4, 5). Later, in 1952, the Iranian Ministry of Health established a nursing division and gradually recognized nursing as a profession (5). Years later and after the Islamic Revolution of 1979, cultural traditions brought about major changes within the higher education system and nursing profession. At this time, nursing education moved from a hospital-based program to a university-supervised system of medical sciences (4). Once Iranian nurses found a new identity, they chose to celebrate Nurses Day on the birthday of Hazard-e Zainab [The daughter of Imam Ali (AS), the first Imam of Shias, who took part in Karbala with her brother, Imam Hossein (AS), the third Imam of Shias to provide care for the injured and sick]. 

Another historic event with significant influence on the Iranian nursing profession was the Iran-Iraq war during 1980-1988, which resulted in nursing advances in terms of clinical, educational, and research development (7). Historical changes in the Iranian nursing profession have presented complex social and ethical issues. According to Vaughans, a historic review of events related to the nursing profession can be traced to sociopolitical and cultural issues of the time, worthy of exploration to create ideas, meanings, and interpretations, and critical to the present and future paths in nursing (8). The history of nursing reveals repeated patterns of encountering professional issues over time and in every decade with new strategies to resolve them. Hence, examination of past events provides effective solutions to future challenges (9). 

The historical research method can link past, present, and future events. Learning about the history of a profession, the process of its development, and its flourishing trends can lead to increased confidence in those associated with the profession. Having historical knowledge of a profession is the cornerstone of professional identity, recognition, and criticism surrounding that profession. Hence, attention to the professional background is necessary. However, as time passes, access to essential resources for deeper understanding of professional identity becomes limited or restricted. Archiving and restoring historical documents are often neglected in developing countries with irreversible losses of learning opportunities. In this study, researchers sought to study and record the history of the Iranian nursing profession and needed access to live witnesses to obtain data from lived experiences and establish records.

The present study describes changes and challenges within the Iranian nursing profession from a sociopolitical perspective. 

## Method

Considering the study objectives, limited access to historical resources and documents, and past library destructions, obtaining oral history from living witnesses of events in Iran provided an opportunity to gain historical reviews and apply the oral history method. The oral history method was first described by Allan Nevins in 1948, and records important events according to recalled lived experiences of study participants. Oral history method refers to the recording of individuals’ verbal statements and is recognized as a scientific and regulated method based on theoretical and practical principles for collecting evidence through verbal documents (10). This method can help produce documents after verification, confirmation, and completion with support of other resources, particularly written documents, and is often used by researchers in the fields of history, sociology, and psychology (11).

To obtain verbal/oral history, 14 senior and experienced nurses in clinical, educational, managerial, and policymaking areas, who live in several provinces of Iran such as Rasht, Mashhad, Tehran, Yazd, Tabriz, Kermanshah, and Urmia, were recruited to serve as the primary data source during September 2013 to November 2014. Shared lived experiences of participants began from 1937 to 2014. Primary sources were obtained from participants through interviews, personal and professional documents, journals, photographs, and letters. The interviews were tape recorded and the researcher took shorthand notes to document each participant’s account in more detail. Secondary sources included newspapers, magazines, relevant articles, books, photographs, governmental documents, and personal archives held at the document conservation centers (the Islamic Revolution Document Center, Library, Museum and Document Center of Iranian Parliament, Central Library of Astan Quds Razavi, and the National Library and Archives of Iran) and medical sciences museums (Tehran University of Medical Sciences and Tabriz University of Medical Sciences, Iran) to support and verify the oral history data. 

The same dramatic stories happened for our documents. Some of the documents were lost by fire or were taken abroad by nurses who left the country after the Islamic Revolution; some other documents were lost or destroyed during pre-Islamic period in the throes of revolution. Moreover, some historic documents were destroyed in previous wars or disposed by the opposing ruling parties. Therefore, we found gaps in the available documents and found it necessary to supplement certain parts of the historical data through interviews and participant recalls.

After obtaining the approval of the Research Deputy of the Ethics Committee at Tehran University of Medical Sciences, the study objectives and procedures were explained to all participants to retrieve signed written informed consent forms. Participant’s interviews lasted 45-90 minutes and took place at a location most convenient to them, which included their homes, offices, or a hotel lobby. 

The oldest living Iranian nurses were selected for participation in this study through purposive sampling. The researchers referred to retirement associations, nursing offices at major universities, and convalescent healthcare facilities to identify suitable participants and interview them. It should be noted that participants were selected to represent a wide variety of past events through maximum variation in age, gender, years of work experience, nursing specialty, professional responsibilities, and place of residence. 

Semi-structured interviews were continued until data saturation was reached. Participants were asked open-ended questions to allow for elaborate responses which were followed by probing questions to further expand on the topic. Examples of open-ended questions ‎used in this study are: “Please tell me about your nursing experience before and after graduation from the nursing school.”, and “Tell me about your work experience as a nurse.” Follow-up and probing questions used were: “According to your past nursing experience…, what seems to have changed within the last few decades and how has the nursing profession evolved?” Exploratory questions and complementary interviews were based on questions such as “Why do you think so?” and “Please explain more.”, to gain a deeper understanding of the participants’ experiences and how they felt about their nursing roles, in order to shed light on the less known aspects of nursing profession in Iran. Data were collected and simultaneously analyzed to form categories and new participants were selected based on data received from previous participants. In total, 17 interviews were conducted with 14 participants for a total of 1,240 minutes. 

**Data analysis**

After the interviews, the recorded data were analyzed using oral history analysis method according to Miller’s four level steps for its applicability to the nursing profession (12, 13). The data analysis method used in this study begins with writing narratives and consists of 4 stages. The first stage involves interviews, that is, collecting oral testimonies related to the study topic. The second stage is the portrait(s) of meaning are presented through biographies for the initial interpretation and reconstruction of interviews in detail according to changes in the social environment as raw data are transcribed verbatim. At this level, similar concepts are grouped and coded to form subcategories and the process of interpreting and validating the historical statements begins, where researcher(s) incorporate secondary data from books, newspapers, and other historical sources to validate their findings. The third stage is the seeds of meaning. Telling extracts are used at this level to focus on finding the key concepts by cross-analysis through validation of biography. Upon the second review, coded data and subcategories are clustered to develop the main categories. At this stage, researchers will be able to identify any inconsistencies by comparing each expression with the whole data pool for cross validation. Rigor of the analysis method is established at the 3^rd^ stage. The fourth stage is the collective meaning; historiography provides the overall picture through a narrative of the researcher’s interpretation derived from the participants' expressions in an integrated story. At the 4^th^ stage, secondary data sources (newspapers, books, articles, and others) are commonly used to better reconstruct the stories. In this study, narratives were used to present the results based on each category and subcategories. 

Researchers focused on a deeper understating of the phenomena through data triangulation, long-term involvement, and data immersion. Date collection and analysis lasted a year from September 2013 to November 2014. External and internal criticism was applied to increase data credibility, verify data genuineness, authenticity, and accuracy. The researchers initially tried to use resources that could verify data validity and if the research team could not validate a resource, the logical deduction was used to confirm or deny the content. After reviewing data and implementing the preliminary content analysis, findings were returned to the participants for confirmation of data accuracy. Moreover, the final versions of narratives retrieved from coded data to develop categories were evaluated by research supervisors and two peer experts in terms of clarity and accuracy. To establish recall related accuracy, individual statements were compared with the whole of the interview and with other interviews, and with the existing historical documents of that time period. These steps activated internal criticism of primary resources. It should be mentioned that participants’ anonymity and confidentiality of the information provided and documents presented were strictly observed throughout the data collection and analysis process. 

## Results

Among the 14 participants, there were 11 women and 3 men, whose ages ranged from 53 to 93 years with the mean age of 67 ± 6 years. Among them, 4 had clinical nursing experience, 4 had managerial experience, and the rest were nurse educators from 7 different cities in Iran. 

We found that within the past century, sociopolitical events had a significant influence on the Iranian nursing profession and researchers were able to identify the 3 main categories of the White Revolution, ‎the Islamic Revolution, ‎and Iran-Iraq war. 

The category of the White Revolution consisted of the subcategories of the Literacy Corps, Health Corps, and gradual formation of the nursing profession. 

The category of the Islamic Revolution consisted of the subcategories of restructuring of higher education, and changing the culture of scientific and administrative systems. 

In addition, the Iran-Iraq war category consisted of the subcategories of the rebirth of nursing, facilitation of the admission of men into the nursing profession, and changing of the curriculum in response to needs.

**The White Revolution**

After the Second World War (WWII), and due to the changes in the western forum, the second Pahlavi king (Shah of Iran) proposed another forum to develop a western paradigm in Iran and enhance its authoritative model under the Pahlavi government. With absolute power, the Pahlavi dynasty, in an attempt to modernize Iran and replace the traditional way of life with a western, and in particular, an American lifestyle, imposed various political and sociocultural changes on the Iranian people and deeply challenged their traditional family values. 

The second Pahlavi king followed his father’s attempts to legitimize power and implement new developments in Iran. To achieve his goals of having a modern society, western ideas were introduced and implemented through 5 socioeconomic programs during 1941 to 1977. In 1948, the Iranian government made changes to the national policies by adding healthcare as the number one priority in a 3-part plan (1948-1967), but no set objectives were presented. The fourth program plan included objectives for preventative health measures such as immunizations and fight against communicable diseases. The fifth program plan focused on the improvement of national health. During 1963, the second Pahlavi king proposed the White Revolution included several codes. Two important components were to develop the Literacy Corps (Sepah-e Danesh) and Health Corps (Sepah-e Behdasht) both of which had significant roles in nursing development. Overall, the White Revolution led to the development of hospitals, mass national education to improve health and education, and increased cultural knowledge. 

*Literacy Corps* (Sepah-e Danesh)

The Literacy Corps, which the second Pahlavi king considered as the capstone among the nine principles, was formed to improve the 20% literacy rate by sending high school graduates, after four months of educational training, to remote villages and small cities to educate people as part of their mandatory military service. Within four years of implementing the White Revolution, university student enrollment rate increased by 18% and the number of hired professors increased by 53% (14). Since the Literacy Crops’ main goal was qualitative and quantitative development of universities and scientific centers, the number of nursing schools and volunteers also increased. 

As part of the White Revolution, there were cultural exchanges between Iran and western countries, particularly with the United States, where thousands of Americans entered Iran and held key positions in order to culturally transition Iran into a western country. They made changes in sociocultural policies through student and scholar exchange programs. They engaged artists and media links between Iran and the United State. 

*[Before the 1960s, most of our physicians came from Europe; for example, Germany, France, and Switzerland. They mostly spoke French and after the White Revolution, things changed. In the 70’s we had more Americans entering Iran…and in those years, nursing programs were integrated from a “License equivalent” (a French term) into a Baccalaureate of Science (B.Sc.) degree by adding more clinical nursing courses to the curriculum. Nursing began to gain social status as a profession and Iranian nurses compared themselves to their American counterparts and performed well.] *(Participant number 14, female, 63 years old, nurse manager)

*Health Corps (Sepah-e Behdasht)*

Health Corps was one of the three principles added to the White Revolution along with the Literacy Corps, which aimed at meeting the national health and education needs. The Ministry of Health increased its budget considerably to achieve the established goals and began building and developing hospitals and healthcare centers, and thus, advancing the nursing profession. The Health Corps recruited a medical team consisting of one physician and two assistants with diploma degrees to treat, educate, and vaccinate people in rural areas (14, 15). Following the creation of the Literacy and Health Corps, public health and literacy levels improved and the number of nursing programs increased. 

*[Another event at that time was having treatment centers managed by oil money. Before that, less than half of a 1000-bed hospital was in operation and after the economic prosperity and increased income from oil money, more treatment centers were built, the 1000-bed hospital was completed and in operation, even in its basement.] *(Participant number 14, female, 63 years old, nurse manager)

*Nursing profession undergoing modernity*

Modernization after the White Revolution included more women entering the nursing profession and families being content with a female dominant job for their daughters. Hence, the Pahlavi dynasty succeeded in improving the professional image of nursing to satisfy the ever-increasing public need for educated nurses. These vast measures helped open more nursing programs during 1956-1971. Thus, the 50’s and 60’s could be considered a prologue for the development of nursing profession in Iran. It should be noted that prior to this era, a small number of nursing programs were dispersed in some parts of the country and they had no integrated curricula. After a few years, some of them closed for various reasons. 

With social changes and the establishment of government-funded national institutions providing nursing training, the 60’s became the era of recognition for nursing as an important profession in healthcare and it was enhanced through public awareness efforts. Moreover, the Ministry of Health increased employment opportunities for nurses, midwives, healthcare technicians (in line with the employment law for nurse, midwife, and technical staff for the Ministry of Health approved on March 03, 1960), and nursing assistants and recognized them as partners in the medical team. Nurses’ and nursing assistants’ salaries increased and the first postgraduate education programs were established at the Pahlavi University in 1967 (5).

*[At that time, my salary as a nurse, working the night shifts, with more difficult tasks, was not any different from that of a school principle. I mean we had a fixed salary without overtime and that was unfair.] *(Participant number 14, female, 63 years old, nurse manager)

The interview results in this study showed an added interest in the nursing profession and subsequent increase in the number of nursing graduates within the next two decades, particularly in the 60’s. Advances in nursing profession encountered new challenges such as lack of nursing instructors, difficult access to educational resources, inadequate educational facilities, and shortage of medical equipment. 

*[At that time, educational facilities were quite scarce. There were few or no books on nursing. Also, there were no modern medical equipment and most tasks were performed manually.] *(Participant number 3, female, 62 years old, instructor)

**The Islamic Revolution**

The Islamic Revolution took place on February, 11, 1979 when the Iranian government structure underwent a fundamental and significant ideological, sociopolitical, and cultural remodeling with the arrival of a new government. These changes were followed by scientific developments and the way nursing was taught, practiced, and later, recognized as a profession. The Cultural Revolution aimed to blend the Islamic ideologies with the academic philosophies at universities by changing the structure of higher education, and subsequently, altering the path to nursing practice.

*Restructuring of higher education*

After the Islamic Revolution, in 1980, the Cultural Revolution Committee under Imam Khomeini’s command authorized the review and modification of academic curricula for all academic majors and the development of new objectives for universities and higher education institutions. One of the major changes occurred in medical sciences with merging of colleges of health sciences with the Ministry of Health (hospitals and healthcare centers). In 1985, medical sciences education was completely removed from the Ministry of Culture and Higher Education, and a new ministry by the name of Ministry of Health and Medical Education was established. This novel ministry mainly focused on health education and medical research by creating a strong network between universities and healthcare facilities to provide health service, treatment, and education, and engage in research in the field of healthcare. This approach centralized nursing education and practice with other medical sciences. 

The nursing branch of the Cultural Revolution Committee was established September 1980 with plans to develop nursing education and offer different degrees in order to meet the nation’s needs in various geographic regions to provide and improve the quality of healthcare services. Prior to the establishment of the Ministry of Health and Medical Education, nursing was mainly a part of medical schools and later separated as an independent profession to promote quality of care in large quantities and meet the Iranians’ public healthcare needs. Since then, more nursing schools have functioned independently, educated and graduated professional nurses, enhanced the quantity and quality of patient care, and improved the public image of nursing as a profession. 

*[In the past, people did not have a proper perception of nursing. But, as time passed and more male and female nurses graduated from college and were employed, the public took notice and viewpoints began to gradually change.] *(Participant number 7, female, 71 years old, clinical nurse and instructor)

The Islamic Revolution followed by the Cultural Revolution, not only changed the educational structure of nursing, but also changed its overall policies including geographical expansion of colleges and their capacities for postgraduate education.

*[One of the factors leading to fundamental changes in nursing education and profession was the student enrolment demand. There were nursing schools in large and small cities competing for students and it was good. The number of postgraduate programs increased to more than what we had in the past. Well educated and experienced nurses helped modify the curriculum with courses in different clinical specialties and changed the culture of nursing education.] *(Participant number 2, male, 62 years old, faculty member)

Immediately after the revolution, there was a greater emphasis on the quantity of nursing programs to graduate more nurses. Meanwhile, the Islamic Revolution had added religious sensitivities to the provision of care regarding gender appropriateness through male and female nurses attending to male and female patients, respectively. Soon noticeable problems surfaced when fewer nurses remained or entered the workforce. This self-imposed shortage of nurses further influenced the quality of care and modified clinical practice and nursing education. 

*[At the beginning of the revolution, instead of quality being taken into account, religious issues became more important and affected nursing education and practice.] *(Participant number 1, female, 63 years old, instructor)

In addition, the Islamic Revolution and political tensions between Iran and Western countries resulted in poor scientific relationships between nursing professionals in Iran and other countries. 

*[At the beginning of the revolution, one of my colleagues and I received academic scholarship to pursue our graduate degree aboard with an acceptable TOEFL score. We applied to 50 colleges and unfortunately none would accept students from Iran.] *(Participant number 8, female, 63 years old, instructor)

*Changing the culture of scientific and administrative systems*

According to the Islamic and Cultural Revolution criteria, acceptance of suitable university professors or students had to abide by the Islamic standards, consisting of appropriate Islamic attire for men and women, having strong faith, and prior sociopolitical involvements in favor of the Islamic Revolution. Individuals who met these requirements were hired and accepted for enrollment in higher education institutes, hospitals, and universities. These extraordinary changes led to the resignation of many nurses and faculty members and their departure from Iran. Immediately after the revolution, healthcare and other systems experienced a great loss of human resources. On the other hand, due to the religious and public acceptance of gender appropriate culture within the universities and healthcare system, the number of female applicants in higher education increased, particularly in the field of nursing. 

 [*One of the factors leading to basic changes in nursing was increased enrollment in universities and more women taking an active role in social activities. This caused a forward public trend.] *(Participant number 2, male, 62 years old, faculty member)

**Iran-Iraq war**

The beginning of the war in 1980 and presence of nurses at the frontlines were a turning point for the Iranian nursing profession, which led to many changes in the way nurses were perceived.

*Nursing rebirth*

With increasing number of nurses serving at the frontline during the Iran-Iraq war, authorities and society at large began to recognize and appreciate nurses. Providing care for injured soldiers in hospitals and caring for the victims of chemical warfare with devastating injuries required extraordinary measures by nurses. 

*[Nurses, particularly men, had a considerable role*

*at the frontline. I, myself took part in eight combat operations when I was a nursing student. We were present to the end and on the scene during the last operation ‘Mersad’. This considerable sacrifice and ever presence at the frontline had a great impact on the Islamic authorities and their viewpoints about the nursing profession.] *(Participant number 4, male, 53 years old, clinician and nurse manager)

 [*With arrival of injured soldiers we did everything we could to improve their condition and people noticed how nurses helped the injured and performed their duties. From then on, the society’s view toward nursing began to change.] *(Participant number6, female, 64 years old, clinical nurse)

*Facilitating admission of men into nursing*

War had a major impact on the Iranian nursing profession and facilitated admission of men into the nursing profession. Before the revolution, few Iranian men were interested in nursing and some of them were educated during their military training. After the Islamic Revolution, enrolling male applicants into a female dominated nursing program was a challenging prospect. More male nurses were needed, especially during and after the Iran-Iraq war. Male nurses found a special opportunity for progress in the nursing profession and were quickly promoted to higher positions even without sufficient training or work experience. Male nursing students were sent to war after completing their basic training and they gained significant first hand clinical experiences at the frontline. Hence, war played an important role in the Iranian nursing education and practice. 

*[At first, men were not easily accepted in nursing programs and later society recognized the need for having male nurses as an important part of the Iranian healthcare system. Men’s entry into nursing was difficult until the pressures mounted during the war and injured male soldiers needed nursing care.] *(Participant number 4, male, 53 years old, clinician and nurse manager)

 [*What helped men find a position in nursing, in my opinion, was that women could not go to the war zones, but men could easily go to the frontline. The Iran-Iraq war made it possible to enroll more men in nursing and reduced hesitation in all aspects toward having male students in a ‘female’ profession.] *(Participant number 2, male, 62 years old, faculty member)

*Changing the curriculum in response to needs*

Nursing education authorities made the necessary changes to meet the nation’s needs by creating a temporary reduction in the number of students pursuing baccalaureate of science (B.Sc.) in nursing and adding to the associate degree nursing (ADN) program in response to the increasing demands for nursing care at the frontline. 

*[The curriculum focus moved from B.Sc. to ADN to help increase the number of trained nurses for hospitals at the frontline. They told us to train technicians to be sent to the hospitals at the war zones, and we added more ADN prepared nurses accordingly… with the stipulation that these nurses could come back after the war and continue their B.Sc.] *(Participant number 4, male, 53 years old, clinician and nurse manager)

## Discussion

This study aimed to review the history of nursing in Iran within a sociopolitical context by interviewing senior nurses who lived through the changes. Developmental progress in nursing is not independent of social events. In this respect, Meleis stated that nursing discipline is a dynamic profession that moves toward novel social changes to meet needs and social demands, albeit, from popular movements, healthcare reforms, or global changes (16). Similar to other performance-based human and social sciences, nursing is not separate from conceptual practices of power, and economic, cultural, and political forces shaping science, philosophy, and knowledge development (17).

Based on the findings of this study, the White Revolution in Iran was one of the most effective ways to help a struggling nation with literacy and health issues. Implementation of Literacy Corps and Health Corps was a step toward the integration of nursing profession in Iran. Governmental supports and the establishment of charitable unions also improved nursing in Iran. With these social progresses and governmental efforts, nursing was introduced as a legitimate profession to the Iranian society. 

Steppe conducted a similar historical study in Germany to examine changes in nursing during the Nazi era and found that German nurses made significant changes in their profession to conform to the Nazi regime (18). At that time, external forces made improvement in social status, unity, and integrity within professional nursing organizations, developed effective nursing laws, and politicized the profession. Moreover, nurses observed ethical and professional principles to provide care without the surrounding social influences. Overall, Steppe argued that advances in nursing could never occur in a neutral and valueless context and are always affected by dominant social forces (18).

In this study, we found that pre-revolution governmental plans, such as the White Revolution during the Pahlavi Dynasty, had a direct influence on nursing education and practice regarding the attraction of girls from higher socioeconomic ranks into the profession. Nonetheless, sociocultural beliefs prevented religious and traditional families from allowing their daughters to pursue nursing.

Another historical and sociopolitical change in Iran was the Islamic and Cultural Revolution, during which the ideologies of Iran’s leadership brought about structural variations in beliefs and nursing values. Kent-Wilkinson stated that social movements, media events, technological progress, and change in public policies can affect training and development, and lead forensic nursing toward a new direction    (19).

In this study, religious and cultural changes showed quantitative and noteworthy improvement in nursing after the Islamic Revolution. Iranian families welcomed the religious and cultural changes after the revolution, and nursing profession succeeded in meeting the nation’s needs for healthcare services. On the other hand, some of the overzealous low-ranking government authorities and instructors imposed rigid requirements upon nursing students and nurses by demanding adaptation to their own ideologies; thus, immediately after the revolution, the quality of nursing education and practice suffered. In this regard, Tabari and Deans stated that one of the main challenges in nursing after the Islamic Revolution was the integration of religious beliefs into the nursing curriculum to meet the society’s spiritual needs through gender-appropriate care (4). As Fooladi reported, nursing programs were gender segregated and the faculty had to divide the curriculum for teaching male and female students in separate sessions, especially in simulation labs and clinical practice (6). To date, such gender sensitivities continue to be regarded and accepted as the Iranian public at large welcome such nursing care provisions through same-gender care; thus, witnessing an increase in male enrolment in nursing (4).

Since religiosity has always been a part of the Iranian culture, the Islamic Revolution simply added a new dimension with a significant effect on women’s rights for higher education, gender-appropriate healthcare and acceptance of men in nursing (6). Overall, the nursing profession in Iran has been positively and negatively affected due to a cyclical path toward religion and cultural sensitivity (20).

With respect to structural changes, transferring of nursing education to universities of medical sciences, gender equality in nursing education, expansion of nursing schools throughout the country, and the development of postgraduate education were among the advances in nursing after the Islamic Revolution. These changes helped form a better social image for the nursing profession due to academic rigor and satisfy cultural beliefs leading to higher student enrolment in nursing. 

Quantitative development of nursing was one of the considerable changes after the Islamic Revolution. The results of a study by Farsi et al. demonstrated that, in recent decades, increase in the number of male nursing students, development of postgraduate education, and introduction of the nursing profession to families improved the social image of nursing (21).

The Iran-Iraq war had a significant effect on the way Iranian nurses performed and later were perceived by the authorities as important healthcare professionals. Cuellar disclosed that in spite of difficulties, nurses used the war as an opportunity to change their image (1). Improved public view towards nursing as well as the need for more men in nursing attracted more male candidates to Iranian nursing programs. During 1985 to 1988, almost 50% of applicants for B.Sc. in nursing were men, and after the war, the number dropped to less than 20% (22).

Being at the frontline was viewed as a religious duty and a personal commitment. Others considered going to war as masculine responsibility to defend the country and help the injured soldiers. In previous studies, it has been reported that wars demanded more male nurses and increased male applicants in nursing programs (23). This does not mean female nurses had any less impact on patient care during wars, because female nurses’ contributions in military hospitals and frontlines remain undeniable. In fact, study findings by Firouzkouhi et al. indicated that during the Iran-Iraq war, female nurses exhibited their advanced clinical and managerial skills (24). For the limited medical supplies, female nurses used their critical thinking skills and offered services while gaining military and clinical experiences (24). 

Peyrovi et al. revealed another aspect of war, by referring to the shortage of human resources after the Islamic Revolution, and social changes exhausting all other resources during the Iran-Iraq war (23). Similarly, we found that the nursing profession responded to national events by changing the curriculum and graduating more ADN instead of B.Sc. nurses to meet the nation’s healthcare needs. 

Nursing has always been responsive to social changes and had a historical relationship with social development, and been determined by religious eras, legacy of wars, and appointed rules to meet patient satisfaction. In each society, health and healthcare ideologies are reflected through welfare and care. Nursing and its developments have been an integral part of any healthcare system.

## Conclusion

This historical review aimed to describe the Iranian nursing profession and examine the sociopolitical events in Iran to find possible influences on nursing. The three major sociopolitical occurrences were the White Revolution, the Islamic Revolution, and the Iran-Iraq war. These events had significant impacts on the Iranian education, healthcare, and nursing profession. Nursing profession showed immense response to sociopolitical events due to having a close relationship with society. Iranian nurses showed professional and personal growth and evolved with new challenges and opportunities.

Development in the Iranian nursing profession did not occur in isolation, and sociopolitical factors had a major influence through mutual understanding between nursing and society. Political decisions and sociocultural movements required changes in healthcare policies to accommodate the new ideological and philosophical structure. Healthcare policies at the governmental levels ultimately modified previous missions with new goals within the nursing profession. 

Events such as wars and Islamic and Cultural Revolution changed the healthcare practice and moved it toward a gender-appropriate system. Moreover, better planning through modifications in nursing curriculum was required to comply with the new rules. Sociopolitical events in Iran played a major role in advancing the nursing profession through policy changes in education, practice, research, and future plans. The nursing profession continues to develop and revise policies to improve the healthcare system and quality of patient care. The results of this study can help in the better understanding and consideration of sociopolitical impacts on the nursing profession when developing new healthcare policies. 

## Limitation

The loss of historical documents and subsequent lack of adequate access to essential resources were the main limitations in this study and were substituted with candid recalls and personal interviews with participants who lived through the previous era and witnessed changes as a professional nurse.
